# Assessment of the Actual Toxicity of Engine Exhaust Gas Emissions from Euro 3 and Euro 6 Compliant Vehicles with the BAT-CELL Method Using In Vitro Tests

**DOI:** 10.3390/ijerph192114138

**Published:** 2022-10-29

**Authors:** Aleksandra Kęska, Anna Janicka, Maciej Zawiślak, Justyna Molska, Radosław Włostowski, Adriana Włóka, Joanna Świeściak, Kacper Ostrowski

**Affiliations:** GEO-3EM Research Centre, Department of Automotive Engineering, Faculty of Mechanical Engineering, Wrocław University of Science and Technology, Na Grobli 13, 50-421 Wrocław, Poland

**Keywords:** actual toxicity, in vitro tests, internal combustion engine, Euro standards, gas chromatography

## Abstract

Legal restrictions on vehicle engine exhaust gas emission control do not always go hand in hand with an actual reduction in the emissions of toxins into the atmosphere. Moreover, the methods currently used to measure exhaust gas emissions do not give unambiguous results on the impact of the tested gases on living organisms. The method used to assess the actual toxicity of gases, BAT-CELL Bio-Ambient-Tests using in vitro tests, takes into account synergistic interactions of individual components of a mixture of gases without the need to know its qualitative and quantitative composition and allows for determination of the actual toxicity of the gas composition. Using the BAT-CELL method, exhaust gases from passenger vehicles equipped with spark-ignition engines complying with the Euro 3 and Euro 6 emission standards were tested. The results of toxicological tests were correlated with the results of chromatographic analysis. It was shown that diverse qualitative composition of the mixture of hydrocarbons determining the exhaust gases toxicity may decrease the percentage value of cell survival. Additionally, it was proven that the average survival of cells after exposure to exhaust gases from tested vehicles meeting the more restrictive Euro 6 standard was lower than for vehicles meeting the Euro 3 standard thus indicating the higher toxicity of exhaust gases from newer vehicles.

## 1. Introduction

The quality of the atmospheric air is determined by different types of pollution sources emitting toxic chemical compounds, which have a negative impact on the health of the human body. In times when the automotive industry is one of the most important trends in the development of society, attention should be paid to the exposure of the human body to the negative effects of such progress. There are many ways to prevent toxins from adversely affecting human health and the environment. In Europe, the basis for the efforts to minimise the harmful effects of exhaust gases from combustion engines on the environment is the imposition of legal restrictions by countries in the form of European exhaust emission standards for commercial vehicles. The tightening of regulations poses a challenge to vehicle designers, who have to ensure that the imposed toxic emission limits are respected. Apart from the necessity to constantly improve vehicles in order to reduce the generation of harmful substances, including changes to the composition of fuel mixtures, increasing the overall efficiency of the engine, the use of exhaust gas treatment systems, and the development of electromobility [1], the intensive development of measurement facilities, which can be costly, is very important. Therefore, the introduction of successive European standards often arouses considerable controversy among both vehicle manufacturers and researchers. These considerations are confirmed by the results of the preliminary research conducted by the authors, which showed that despite the tightening of emission standards and appropriate structural modernisation in the powertrain system, the emission of toxic compounds into the atmosphere has not decreased [2].

Currently used methods of exhaust gas emission testing imposed by legal standards consist of measuring concentrations of particular components of exhaust gases [3,4,5,6,7,8,9]. However, these methods do not take into account mutual interactions between particular compounds that are cumulative or can intensify or cancel each other’s toxic effects. Therefore, they do not give a direct, clear result about the toxicity of the gases or the negative impact on living organisms. It seems reasonable to use a method that makes it possible to determine the actual impact of toxic substances on a living organism in a relatively quick, unambiguous and objective way. Actual toxicity refers to the harmful effects of a given substance on living organisms—tissues, organs or biological processes. Toxicological methods seem to be suitable for this purpose. However, the methods of assessing gas mixture toxicity used so far—indirect (computational) and direct (in vivo, in vitro)—have many disadvantages (Section 1.1). However, the introduction of innovative methods of assessment of gas mixture toxicity using in vitro tests has eliminated many of their shortcomings. The precursor of this type of testing comes from the German company, Vitrocell, and deals with the exposure of living cells to the effects of pollutants inhaled by humans [10,11]. The BAT-CELL Bio-Ambient-Tests method was developed, inter alia, for testing vehicles—toxicity of cabin air and engine exhaust gases [12,13,14].

The BAT-CELL method is based on an assessment of the influence of the actual toxicity of various gas mixtures on living cells taking into account additive synergism. It allows for direct contact between the tested gas and the cell surface, thanks to the elimination of the physicochemical barrier in the form of culture fluid, which distinguishes it from other direct methods of this type. Direct exposure of cells to the tested gases, using the BAT-CELL device, is a reproducible technique for testing the cytotoxicity of gas mixtures [15]. The method allows tests to be carried out on immission and emission in a diverse range of facilities, at workstations and in indoor environments [16,17,18]. In the present study, this method was applied to investigate the actual toxicity of engine exhaust gases.

In order to find a correlation between the qualitative and quantitative composition of the tested gas mixture and the actual toxicity of engine exhaust gases, the study also used the gas chromatography method. This method allows for the identification of the composition of hydrocarbons contained in the tested gas mixture, which determines the toxicity of exhaust gases. In this group of compounds, volatile organic compounds (VOCs) and polycyclic aromatic hydrocarbons (PAHs) deserve special attention due to their carcinogenic and mutagenic properties [19,20,21,22,23]. Therefore, it was decided to compare these groups of compounds with the exhaust gas cytotoxicity results.

### 1.1. Toxicity Assessment Methods

Methods for assessing the toxicity of gas mixtures can be divided into indirect (computational) and direct methods. Indirect methods are computational indicators based on legal regulations and they are determined experimentally. Direct methods are in vivo methods (tests inside a living organism) and in vitro methods (tests on living cells isolated from the organism).

The advantages of calculation methods include the ease of their application and, generally, the lack of complex calculations. Calculation indicators based on legal regulations are commonly used in environmental reports and environmental impact assessments. 

The disadvantage of the indirect methods is the need to know the qualitative and quantitative composition of the mixture, as its absence usually disqualifies the method. Furthermore, these methods do not take into account the synergistic effects associated with the presence of several compounds simultaneously. Regulatory-based indicators cannot be used when an unstandardised component is present in the mixture. Changes to the applicable reference standards lead to changes in the values of the indicators. Experimentally determined indicators sometimes require the use of very complex computational algorithms.

The advantage of the direct methods is undoubtedly the possibility to study the direct influence of a given mixture on a living organism. It is also not necessary to know the qualitative and quantitative composition of the mixture. In addition, in vitro tests exclude animal suffering and do not require the consent of the bioethics committee. 

The disadvantage of in vivo testing is that it is primarily a complicated and time-consuming path requiring many tests and leading to an expected, unambiguous result. It is also difficult to isolate a single source of exposure. In addition, study results may be influenced by the characteristics of the species. Such studies involve animal suffering and require the consent of the bioethics committee.

So far, in in vitro studies, the problem has been the correct determination of exposure parameters, such as the time and manner of contact with the gas mixture. In addition, the presence of the culture fluid in which the test cells arrived has been a barrier preventing the cells from coming into direct contact with the tested gas mixture. In other words, the qualitative and quantitative characteristics of substances reaching the cell layer, i.e., the dose causing the toxic effect, have not been known. Due to the sensitivity of cell lines, all physicochemical factors affecting cell viability should be eliminated as far as possible.

The disadvantages of living cell studies to date have largely been eliminated thanks to the patenting of innovative methods for assessing the toxicity of gas mixtures using in vitro tests [10,11,12,13,14].

### 1.2. In Vitro Studies

Analysis of the literature sources covering the last few years does not find that researchers have used any newer, improved methods than the BAT-CELL method. For years, the principle of the technique used in this area has not changed fundamentally.

Studies on living cells are carried out on the basis of a number of different, commonly used tests to assess cell cytotoxicity using: changes in cell membrane integrity, changes in lysosome activity, activity of enzymes involved in cell metabolism, ability of the cell to divide, i.e., proliferation, and activation of programmed cell death pathways. Selected publications describing the research conducted with the use of in vitro methods are presented below.

A Greek researcher, Velali, is the author of many available papers that describe the results of in vitro and in vivo toxicological tests [24,25,26,27]. The samples were usually gases contained in the air of urban agglomerations (city centres and the outskirts) or gases emitted from residential heating systems and exhaust gases from SI (spark ignition) and CI (compression ignition) engines. Velali’s biological studies are based on cytotoxicity testing using the MTT test (3-(4,5-dimethylthiazol-2-yl)-2,5-diphenyltetrazolium bromide; based on mitochondrial oxidoreductive activity, assuming that the MTT dye is reduced only in living cells) and LDH (lactate dehydrogenase; using an enzyme that moves from the cell to the culture medium due to changes in cell membrane integrity) and genotoxicity testing based on in vivo tests: SCE assay (sister chromatid exchange test) and comet assay (examining damage to DNA structure and relying on the method of cell electrophoresis in agarose gel). Velali’s work also uses the well-known Ames test for detecting mutagenicity, in which indicator bacterial strains are tested. Other authors have conducted similar studies on urban air pollution in their work, using both the most popular MTT test [28] and LDH [29] as well as, for example, the Alamar Blue test [30,31]. 

Czerwiński is a Polish author of papers, published in Switzerland, based on studies of living cells exposed to gas mixtures [29,32]. Czerwiński’s recent papers describe the adverse biological effects of volcanic dust and exhaust gases from a petrol engine on the A549 cell line [32]. The author’s other papers show the results of cellular cytotoxicity studies of engine exhaust gases from vehicles with a GDI (Gasoline Direct Injection) engine equipped with a gasoline particulate filter (GPF) and without a filter. The effects of the addition of high and low ash oil were also investigated [29].

The key issue, however, is not selecting the right test, but selecting the right parameters for cell exposure to harmful substances and knowing the dose of tested gases causing the toxic effect. Nonetheless, in all of the above studies, cell cultures were kept in culture fluid in an incubator at a constant temperature of 37 °C and a concentration of 5% CO_2_ for up to 24 h of exposure. 

Numerous scientific papers on actual gas toxicity refer to cigarette smoke toxicity [33,34,35,36,37]. These studies are based on in vitro tests and mainly compare different types of cigarettes or e-cigarettes in terms of smoke toxicity.

The most recent studies from 2021 based on in vitro tests describe, inter alia, studies of CO_2_ absorption by seeds and plant pods in the context of greenhouse gas emissions [38] or toxicity studies of different types of fuels with the use of biomarkers [39].

On the basis of the literature review, it can be concluded that the actual toxicity of gas mixtures is an important, topical issue that requires further research. The aim of this study is to develop the BAT-CELL method for testing the actual toxicity of engine exhaust gases.

## 2. Materials and Methods

### 2.1. Selecting Vehicles for the Study

Due to the market share trend and the quality composition of engine exhaust gases (mainly variation in the hydrocarbon composition), the decision was made to opt for vehicles equipped with a spark-ignition engine. Such a choice also seemed to be more scientifically interesting because current research on exhaust emissions is mostly concerned with vehicles with CI engines [40] even though currently the technological development of vehicles with SI engines is equally dynamic. Exhaust gas samples were taken from six vehicles equipped with a spark-ignition engine: three meeting the Euro 3 standard and three meeting the Euro 6 standard (Table 1). The choice of emission standards was guided by the various degrees of development of exhaust gas treatment technologies applied in the vehicles. 

The vehicles to be tested were selected at random. Each of them had a valid technical inspection conducted at a vehicle inspection station. Additionally, a vehicle checking procedure was carried out, which unequivocally disqualified vehicles with leaks in the exhaust system, erratic engine operation, engine controller errors, faulty cooling systems, incomplete exhaust systems, or leakage of operating fluids. All the petrol units tested were equipped with the same components of the exhaust gas treatment system, i.e., exhaust gas recirculation systems and three-way catalytic converters (only vehicle F was additionally equipped with a GPF). Before starting the exhaust gas sampling, the catalytic converter in each vehicle was heated to operating temperature. 

### 2.2. Method of Exhaust Gas Sampling

The exhaust gases were sampled at idle while controlling the hydrocarbon (HC) concentration value with a Horiba Mexa—584L exhaust gas analyser. When the engine runs at the correct temperature, the emission of harmful exhaust gas components should be relatively low. When the temperature is lower than the correct one, only a part of the supplied fuel is burnt and thus the HC level in the exhaust gas increases. 

The exhaust gases were sampled using an aspirating system, in which an inert gas-sampling bag was placed and connected with a silicon tube to the vehicle exhaust pipe. The exhaust gases were collected from each vehicle into five pre-prepared inert sampling bags. The bags were filled in a certain sequence. First, the bags for the subsequent collection of gases for activated carbon in order to test VOC (1 bag) and PAH (3 bags), then a bag for gases to which living cells would be exposed (1 bag).

The exhaust gases from the sampling bags for activated carbon were collected using the ASP 3 II automatic aspirator with a flow rate set at 30 dm^3^/h. Samples with activated carbon were stored at a temperature below 20 °C until they were submitted to laboratory tests for the presence of VOCs and PAHs.

### 2.3. The BAT-CELL Bio-Ambient-Tests Method

The BAT-CELL method enables the measurement of toxic effects of gas mixtures on human health using a suitable cell line dedicated to toxicity tests. The selected cell culture—a continuous line of L929 fibroblast-like cells obtained from mouse subcutaneous tissue [42] devoid of culture fluid—was placed in a sterile closed sampler. Then, exhaust gases were injected into the sampler with an inlet tube through an antibacterial filter and with the help of the aspirating system. After the cell culture had been exposed to the tested gases, it was flooded with culture fluid and the toxic effects of the exhaust gases on the culture were examined using a standard toxicological test—flow cytometry identifying the fluorescence intensity of the cells. Test results were automatically given as a percentage of cell survival. The exposure time was chosen individually for the type of gas mixture tested. For engine exhaust gases it was 7.5 min [2]. The flow parameters were adjusted to the shape of the sampler in such a way as to enable uniform contact of the gas particles with the cell surface and so as not to damage them mechanically. The flow rate of the tested gases through the aspirating system was set at 150 cm^3^/min.

The conditioning chamber in which the samplers are placed is equipped with pressure and temperature sensors to ensure that the vital functions of the cell culture are maintained. The elimination of culture fluid is possible by maintaining physical parameters appropriate to the requirements of a given cell line, safe for the timeline of the cells outside the incubator atmosphere and without the supply of nutrients. The culture fluid also performs an antibiotic function for cells; thus, the cell line sampler is additionally protected at the inlet with an antibacterial filter [13].

Three types of control trials were carried out in the research in order to eliminate errors in the measurement procedure. A control test was performed, in which the sampler with cells was left in the culture fluid in a CO_2_ incubator in the laboratory. Moreover, an inspection was carried out to eliminate errors resulting from flow disturbances in the aspiration system. For this purpose, ambient air was passed through three samplers with cells without culture fluid. The control was also performed by leaving the sampler with cells in the BAT-CELL chamber without the culture fluid and without connecting the aspiration tubes.

### 2.4. Gas Chromatography

Gas chromatography is the only available method for qualitative and quantitative assessment of the hydrocarbons present in a given gas mixture. The compounds were determined in accordance with the internal procedures of the laboratory performing the analysis. 

The Varian GC-450 gas chromatograph with a flame ionization detector and a capillary column (Varian VF-WAXms 30 m × 0.25 mm ID DF: 0.25 μm) was used to determine VOC. Carbon disulfide was used as the extractant. The work was carried out at a fixed column temperature of 373 K (110 °C) using a 423 K (150 °C) dispenser and 423 K (150 °C) detectors.

The determination of PAHs was performed with the use of a mass detector (MS). The method uses the SPE solid phase extraction technique. A C18 chromatography column with the smallest possible particle size and porosity was used. In the case of PAHs, dichloromethane was used as the extractant. For qualitative and quantitative analysis, an Agilent Technologies 7890B gas chromatograph coupled with a 5977A MSD mass spectrometer from the same company with an automatic feeder and an appropriate capillary column (DB-EUPAH) was used. The following oven temperature program was used: 45 °C (0.8 min), temperature increase from 45 °C/min to 200 °C, temperature increase from 2.55 °C/min to 225 °C, temperature increase from 3 °C/min to 266 °C, then temperature increase from 5 °C/min to 300 °C (5.289 min). Analysis time: 40 min.

Before starting the analysis of VOC and PAH, the devices were calibrated and the recovery of methods was determined by determining the calibration coefficients and desorption coefficients. The total relative error of the VOC analysis method was estimated at 20% and at 30% for PAH.

## 3. Results and Discussion

### 3.1. Actual Toxicity of Exhaust Gases

**Figure 1:**
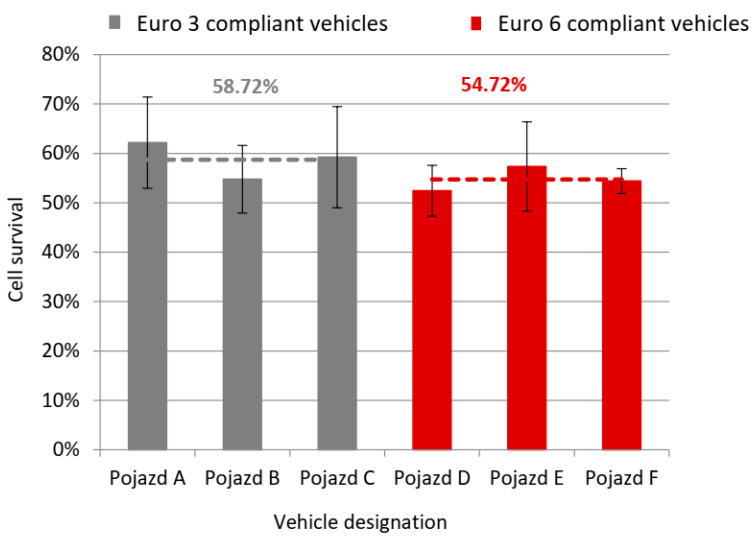
Mean cell survival calculated with the flow cytometry method.

The graph (Figure 1) shows the average survival rate of cells after exposure to engine exhaust gases of individual vehicles. The dotted line indicates the average cell survival rate for vehicles meeting the Euro 3 standard, 58.72%, and the Euro 6 standard, 54.72%. The results showed that the cell survival rate for vehicles complying with the more restrictive Euro 6 standard was 4% lower than that of Euro 3 compliant vehicles. The mean cell survival rate for each vehicle was calculated from three trials. The mean standard deviation error for Euro 3 compliant vehicles was 8.78% and for Euro 6 compliant vehicles, 5.57%. In order to check the statistical significance of differences in the values of cell survival for the two groups of vehicles, Student’s t-test was performed for independent samples. The value of the t-Student statistics was 0.876. For 4 degrees of freedom, the significance level was 0.43. The critical value read from the distribution table was 0.7407 and was lower than the determined value of the Student t-statistic. This means that the level of significance of the results is lower than that assumed in the table, and therefore the difference in the mean cell survival values for both groups of vehicles is not statistically significant. However, this does not mean that the results are not significant at all.

In the study, the probability of cell death due to external factors (stress, physical, mechanical) was minimised. The total cell count/mL for all trials performed for a single vehicle should vary by an order of magnitude at most, which was maintained. Furthermore, the total cell count/mL did not decrease after the exposure of cells to the tested gases.

The cell culture was observed under the microscope before exposure, directly after exposure, and after 24 h, and the cells were counted after 48 h. The control microscope image clearly showed a large number of cells compared to the microscopic image of the cells after 48 h exposure to the exhaust gas.

During the tests, control tests were carried out, the results of which confirmed the correctness of the tests. The mean cell survival rate after exposure to ambient air was 75.25%. This result may have been due to the oxygenation of the cells that were supplied with clean air during the exposure. The results of the control tests without the medium flow were comparable with each other. For the control sample left in the laboratory, cell survival was 65.85%, and for the sample located in the BAT-CELL chamber, it was 65.60%.

The lower mean cell survival for vehicles meeting the more restrictive Euro 6 emission standard is mainly due to differences in technology and design used in all tested vehicles. All the petrol units tested were equipped with the same components of the exhaust gas treatment system. Although the principle of operation of these components is similar, due to the development of manufacturing technology and the use of different construction materials, they may have been responsible for different levels of emission of toxic compounds. As is well known, many construction materials are responsible for the emission of volatile organic compounds [18,43]; whereas, the use of precious metal catalytic layers is responsible for the emission of harmful by-products, including nitrogen dioxide, sulphates or ammonia [21,44,45,46,47,48].

### 3.2. Chemical Analysis of Hydrocarbons Contained in Exhaust Gases

Hydrocarbons, as a group of compounds, are subject to emission limits. They include, in relatively small amounts, volatile organic compounds, which are often highly toxic, and polycyclic aromatic hydrocarbons, which are not directly limited but which account for the toxicity of the whole group of hydrocarbons. 

#### 3.2.1. Volatile Organic Compounds

Table 2 shows the results of the concentration values of the particular VOC groups: aromatic hydrocarbons, paraffinic hydrocarbons and alcohols, as well as the values of the sum of VOC concentrations for individual vehicles.

The highest values of VOC concentrations were recorded for vehicle C, followed successively by E and B. Analysis of the results showed that the average percentage of aromatic hydrocarbons in the total VOC group for vehicles meeting the more restrictive Euro 6 standard (19.3 mg/m^3^) was higher than that of vehicles meeting the Euro 3 standard (17.7 mg/m^3^).

**Figure 2:**
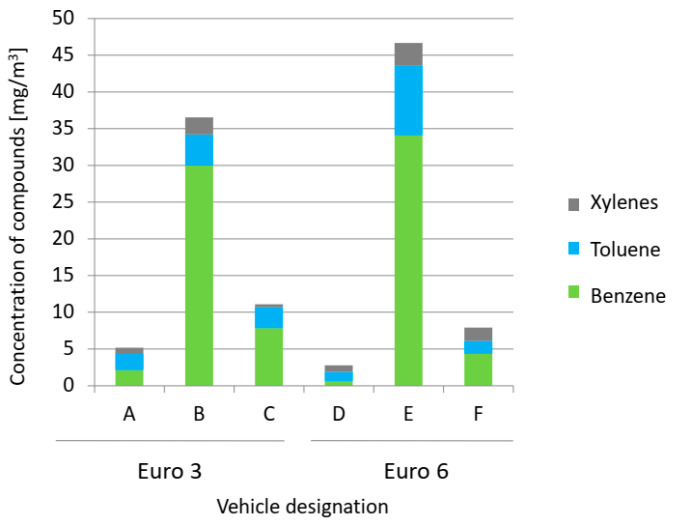
Concentration of BTX group (benzene, toluene, xylenes) compounds.

Compounds from the group of aromatic hydrocarbons were analyzed, including compounds from the BTX group (benzene, toluene, xylenes) and ethylbenzene, cumene, propylbenzene, mesitylene and p-cymene. The highest value of BTX concentration (Figure 2), including for benzene (34 mg/m^3^), toluene (9 mg/m^3^) and xylenes (3 mg/m^3^), was recorded for vehicle E (46 mg/m^3^) and vehicle B (37 mg/m^3^). For the remaining vehicles, these values were below 11 mg/m^3^. The presence of benzene, toluene and xylenes (p-xylene, m-xylene, o-xylene) was recorded for each vehicle.

**Figure 3:**
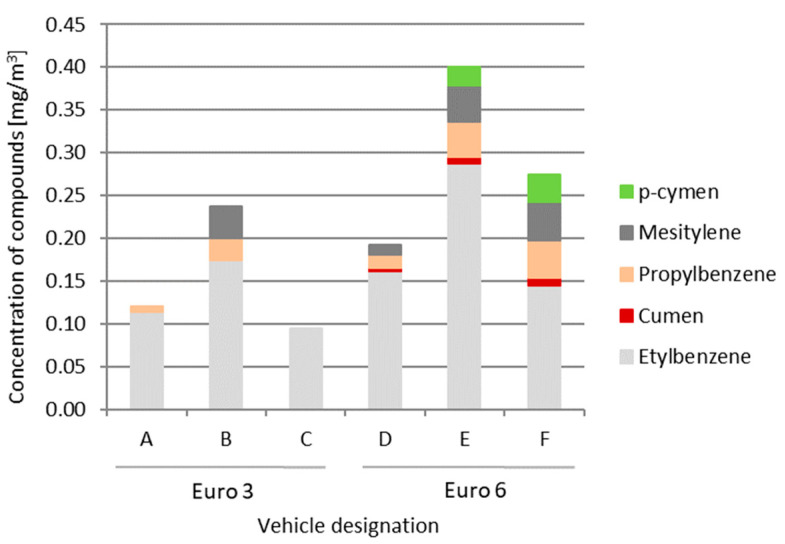
Quantitative assessment of aromatic hydrocarbon compounds (BTX omitted).

For Euro 6 vehicles, the variety of compounds is greater than for Euro 3 vehicles (Figure 3). Cumene and p-cymene are not found in older vehicles compliant with the Euro 3 standard. Propylbenzene and mesitylene are also compounds found more frequently in the Euro 6 group. The highest concentration of compounds from the aromatic hydrocarbons group was recorded in vehicle E (47 mg/m^3^). The values were several times higher than those of other vehicles.

**Figure 4:**
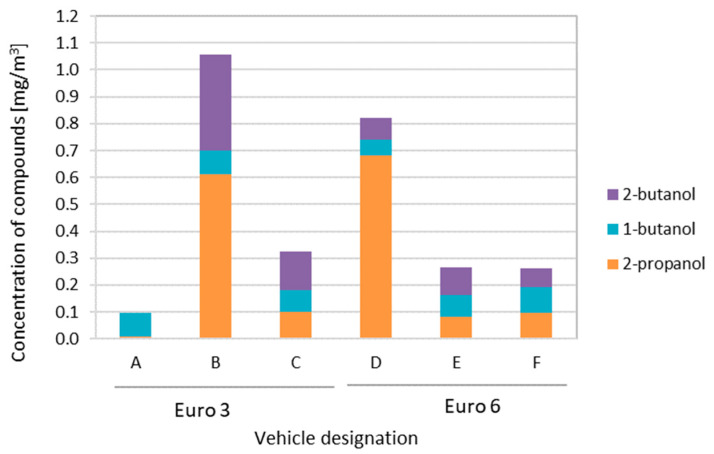
Quantitative assessment of alcohols group.

Three different compounds were detected among the alcohols (Figure 4). The highest total concentration of alcohols was recorded for vehicles B and D. In the group of paraffinic hydrocarbons for each of the vehicles, the presence of one compound—n-pentane—was recorded.

#### 3.2.2. Polycyclic Aromatic Hydrocarbons

Table 3 shows the PAH concentration values for individual vehicles. Among vehicles meeting the Euro 3 standard, the highest values were recorded for vehicle B—4691 µg/m^3^. On the other hand, among the newer models (Euro 6), the highest PAH concentrations were recorded for vehicle D—881 µg/m^3^. In total, half of the tested vehicles had elevated PAH concentrations, of which two of the vehicles were of Euro 6 standard and one was of the Euro 3 standard.

**Figure 5:**
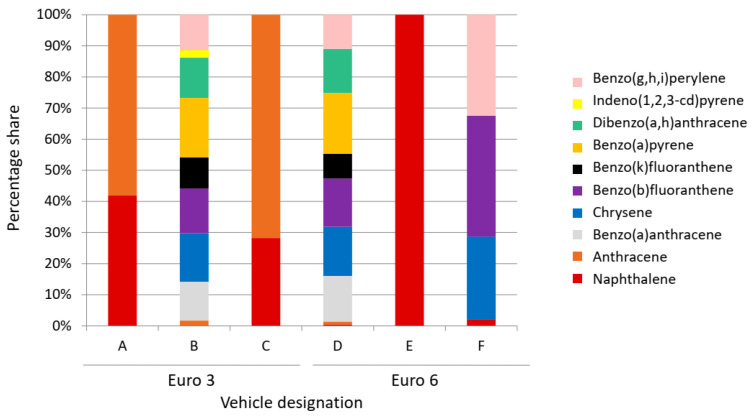
Qualitative assessment of the PAH group compounds.

For vehicles B and D, benzo(a)pyrene accounted for the largest share of PAH emissions and only for these two vehicles was its presence recorded (Figure 5). Although benzo(a)pyrene is treated as an indicator of PAH air pollution, the presence of many other toxic compounds was noted in the exhaust gases of half of the vehicles studied. In vehicle F, no benzo(a)pyrene was registered, but the presence of chrysene, benzo(b)fluoranthene and benzo(g,h,i)perylene was recorded. In the whole group of PAH compounds for vehicle E, only one component was determined—naphthalene. For vehicles A and C, naphthalene and anthracene were identified. For vehicles B and D, the greatest variety of compounds present was recorded.

The qualitative assessment of compounds from the PAH group showed that benzo(a)pyrene, which is recognised as a determinant of the concentration of the polycyclic aromatic hydrocarbon group, was identified in only two vehicles and did not account for the dominant percentage of the whole group of compounds. The diverse qualitative-quantitative composition of compounds from the PAH group for individual vehicles may be caused, for example, by the operation of a catalytic converter, e.g., wear and tear of components during their operation.

### 3.3. Correlations

Interpretation of the study results is based on comparison of the actual toxicity of the engine exhaust gases and the qualitative and quantitative composition of the hydrocarbons emitted from the two groups of vehicles complying with the Euro 3 and Euro 6 standards, respectively. There are no indicators of the degree of actual toxicity depending on cell survival or compound concentration. There is no toxicity scale that would indicate the level of actual toxicity of a given gas mixture.

Analysis of actual exhaust gas toxicity results obtained through flow cytometry revealed a 4% difference in mean cell survival between the two groups of vehicles compliant with the Euro 3 and Euro 6 standards (Figure 3). The higher survival rate was found in the Euro 3 group of vehicles. Lack of statistical significance of this difference does not change the fact that the obtained values do not meet the expectations—there was no significant reduction of exhaust gas toxicity. Looking at the changes in emission limits that have taken place over the years, it may be observed that the values of maximum allowable concentrations are more than twice lower for the Euro 6 standard as compared to the Euro 3 standard. The percentage decrease in the allowable concentration values of the limited compounds observed between the introduction of the Euro 3 and Euro 6 standards is 56% on average (Table 4). The table presents only those groups of compounds for which values exist and can be compared.

The comparison shows that the reduction of allowable concentration values of particular compounds in engine exhaust gases does not translate into a reduction of the actual toxicity of the gases studied.

**Figure 6:**
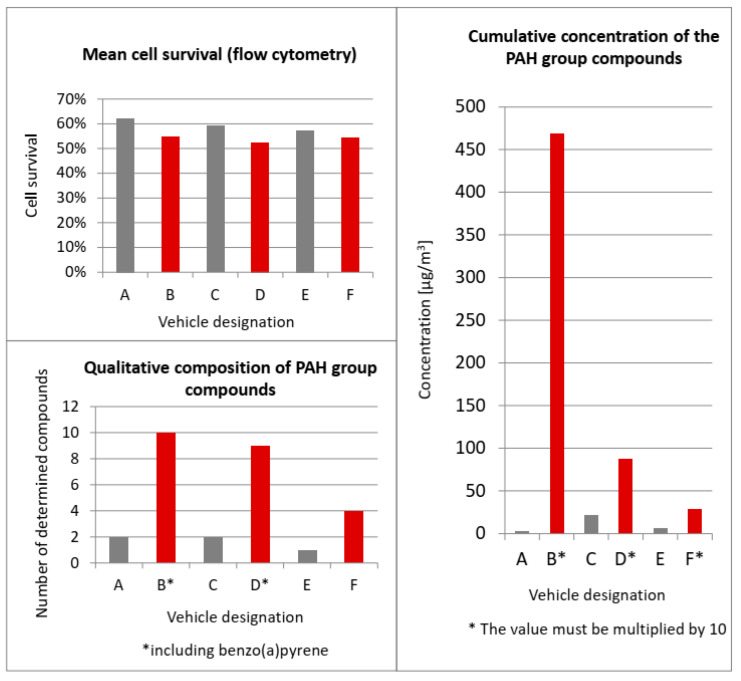
Correlations between the values of percentage cell survival and the number and cumulative concentration of compounds determined in the PAH group.

Considering the mean cell survival rate and the qualitative and quantitative composition of compounds from the polycyclic aromatic hydrocarbon group, certain regularities can be observed. Relevant graphs are presented to illustrate the occurring correlations (Figure 6). For vehicles B, D and F, the lowest values of percentage cell survival and the highest concentration values of compounds from the PAH group were recorded, as well as the highest number of compounds determined in this group (marked in red in the tables). For vehicles B and D, the presence of benzo(a)pyrene in the exhaust gases was identified and it was for these vehicles that the lowest cell survival rates were recorded.

On the basis of HC content in exhaust gases, the share of polycyclic aromatic hydrocarbons (PAHs) in the entire group of HC compounds was calculated (Table 5). For this purpose, the values of hydrocarbon (HC) concentrations in exhaust gases, obtained for individual vehicles with the use of an exhaust gas analyser, were converted from ppm to µg/m^3^ units. The mean HC value from the three measurement points (points 2–4) was taken into account, as the exhaust gases collected at that time that were used to determine PAHs.

**Figure 7:**
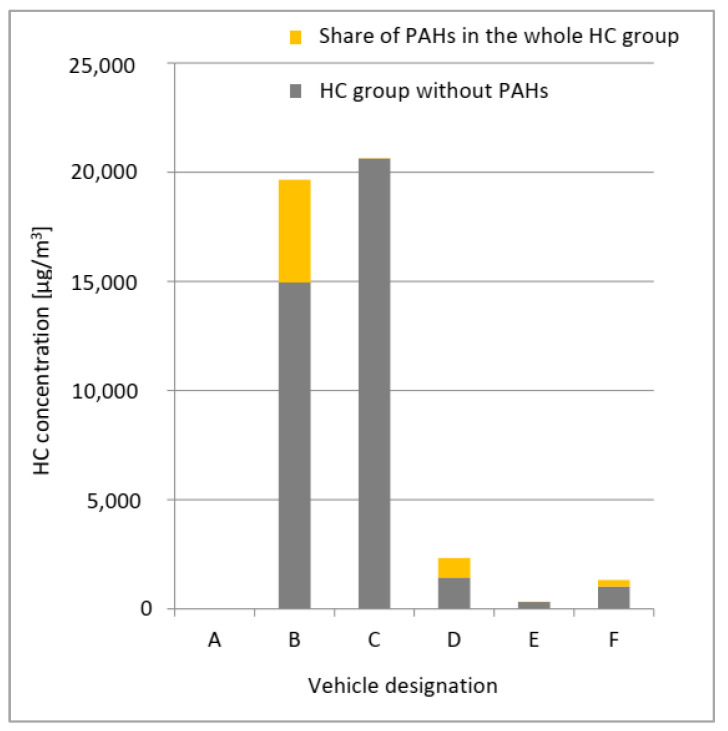
Share of polycyclic aromatic hydrocarbons (PAHs) in the hydrocarbon (HC) group.

On the basis of the calculated values, a graph (Figure 7) was prepared demonstrating the share of PAHs in the whole group of hydrocarbons (HC). 

For vehicle A, no increase in HC was observed during the operation of the engine. Considering the very low PAH concentration value for vehicle A, it can be concluded that the exhaust gas analyser used had insufficient measurement accuracy. The highest share of PAHs in the whole hydrocarbon group was recorded for vehicles B, D and F. Vehicle C, despite high concentrations of the entire hydrocarbon group, did not contain a significant amount of polycyclic aromatic hydrocarbons.

The presented analyses show that it is likely that lower cell survival may be associated with elevated concentrations of compounds from the polycyclic aromatic hydrocarbon (PAH) group and/or the diverse qualitative composition of compounds in this group. A large number of determined compounds in the PAH group are associated with a series of interactions between individual substances (synergistic effect), which may result in the increased toxicity of the entire mixture of gases that constitute the studied engine exhaust gases. In addition, the presence of benzo(a)pyrene, as a compound showing the most toxic properties among the PAHs, may intensify the toxic effect. Therefore, it should be borne in mind that it is the qualitative and quantitative composition of compounds from the PAH group that can determine the actual toxicity of exhaust gases, and it is this group of compounds that should perhaps be subjected to detailed analysis during emission tests when approving vehicles for use on public roads as an additional type-approval test.

On the basis of this study and the analysis of all the results, it can be concluded that the BAT-CELL Bio-Ambient-Tests measurement method used is relatively simple both in terms of conducting the experiment and interpreting the results. In comparison with other methods used, apart from taking into account the synergistic effect, the BAT-CELL method does not require knowledge of the qualitative and quantitative composition of the gas mixture, nor is it dependent on calculation indicators dependent on changing legislation. Therefore, this method could be an alternative to existing methods for the assessment of exhaust gas toxicity.

## 4. Conclusions

The conducted research allowed for the application of the innovative BAT-CELL method based on in vitro tests to test the actual toxicity of exhaust gases. Thanks to the development of the new method, it will be possible to use it as an alternative to assessing the quality of exhaust gases from internal combustion engines. The tested vehicles showed a higher toxicity of exhaust gases for newer units that meet the Euro 6 standard. This condition may be caused by significant differences in the qualitative and quantitative composition of compounds from the group of aromatic hydrocarbons in the exhaust gases of the tested vehicles. Thanks to unambiguous and clear results of toxicological tests (percentage cell survival) it is possible to increase the awareness of vehicle users about the real threat posed to their own health by the improper operation of exhaust system components or their removal (e.g., catalytic converter).

## Figures and Tables

**Table 1:** Test vehicles data [41].

Euro Standard	Euro 3			Euro 6		
Vehicle identification	A	B	C	D	E	F
Year of production	2004	2002	2003	2018	2019	2018
Type of bodywork	hatchback	hatchback	kombi	kombi	sedan	hatchback
Engine capacity [cm^3^]	1998	1598	1390	1798	2488	999
Engine power [kW]	99	55	55	132	141	85
Mileage [thou. km]	236	166	227	45	33	30

**Table 2:** Concentrations of the particular VOC groups in the tested vehicles.

VOCs Concentration [mg/m^3^]	Euro 3			Euro 6		
	Vehicle A	Vehicle B	Vehicle C	Vehicle D	Vehicle E	Vehicle F
Aromatic hydrocarbons	5	37	11	3	47	8
Alcohols	0	1	0	1	0	0
Paraffinic hydrocarbons	4	45	132	1	64	1
Total VOC	9	83	143	5	111	9

**Table 3:** PAH concentration in the tested vehicles.

	Euro 3			Euro 6		
	Vehicle A	Vehicle B	Vehicle C	Vehicle D	Vehicle E	Vehicle F
PAH concentration [µg/m^3^]	3	4691	21	881	6	291

**Table 4:** List of the decreases in mean allowable concentration values of limited compounds and cell survival rates for tested Euro 6 vehicles compared to Euro 3 vehicles.

Group of Compounds	Euro 3	Euro 6	Decrease in Allowable Concentration Values of Limited Compounds	Mean Decrease in Allowable Concentration Values of Limited Compounds	Mean Decrease in Cell Survival Values
CO [mg/km]	2300	1000	57%	56%↓	4%↓
HC [mg/km]	200	100	50%		
NO_X_ [mg/km]	150	60	60%		

**Table 5:** The HC and PAH contents in engine exhaust gases.

Vehicle Designation	HC [ppm] (Mean of Measurement Points 2–4)	HC [µg/m^3^] (Mean of Measurement Points 2–4)	PAH [µg/m^3^]
A	-	-	3
B	59	19,647	4691
C	62	20,646	21
D	7	2331	881
E	1	333	6
F	4	1332	291

## Data Availability

Data is contained within the article.

## Abbreviations

HC, hydrocarbon; PAH, polycyclic aromatic hydrocarbon; VOC, volatile organic compound
